# Regulation of poly(ADP-ribose) polymerase-1 (PARP-1) gene expression through the post-translational modification of Sp1: a nuclear target protein of PARP-1

**DOI:** 10.1186/1471-2199-8-96

**Published:** 2007-10-25

**Authors:** Karine Zaniolo, Serge Desnoyers, Steeve Leclerc, Sylvain L Guérin

**Affiliations:** 1 Oncology and Molecular Endocrinology Research Center, Centre de Recherche du CHUL-CHUQ and Département d'Anatomie-Physiologie, Université Laval, Québec, G1V 4G2, Canada; 2 Unité de Recherche en Pédiatrie, Centre de Recherche du CHUL-CHUQ and Département de Pédiatrie, Université Laval, Québec, G1V 4G2, Canada

## Abstract

**Background:**

Poly(ADP-ribose) polymerase-1 (PARP-1) is a nuclear enzyme that plays critical functions in many biological processes, including DNA repair and gene transcription. The main function of PARP-1 is to catalyze the transfer of ADP-ribose units from nicotinamide adenine dinucleotide (NAD^+^) to a large array of acceptor proteins, which comprises histones, transcription factors, as well as PARP-1 itself. We have previously demonstrated that transcription of the PARP-1 gene essentially rely on the opposite regulatory actions of two distinct transcription factors, Sp1 and NFI. In the present study, we examined whether suppression of PARP-1 expression in embryonic fibroblasts derived from PARP-1 knockout mice (PARP-1^-/-^) might alter the expression and/or DNA binding properties of Sp1 and NFI. We also explored the possibility that Sp1 or NFI (or both) may represent target proteins of PARP-1 activity.

**Results:**

Expression of both Sp1 and NFI was found to be considerably reduced in PARP-1^-/- ^cells. Co-immunoprecipitation assays revealed that PARP-1 physically interacts with Sp1 in a DNA-independent manner, but neither with Sp3 nor NFI, in PARP-1^+/+ ^cells. In addition, *in vitro *PARP assays indicated that PARP-1 could catalyze the addition of polymer of ADP-ribose to Sp1, which also translated into a reduction of Sp1 binding to its consensus DNA target site. Transfection of the PARP-1 promoter into both PARP-1^+/+ ^and PARP-1^-/- ^cells revealed that the lack of PARP-1 expression in PARP-1^-/- ^cells also results in a strong increase in PARP-1 promoter activity. This influence of PARP-1 was found to rely on the presence of the Sp1 sites present on the basal PARP-1 promoter as their mutation entirely abolished the increased promoter activity observed in PARP-1^-/- ^cells. Subjecting PARP-1^+/+ ^cells to an oxidative challenge with hydrogen peroxide to increase PARP-1 activity translated into a dramatic reduction in the DNA binding properties of Sp1. However, its suppression by the inhibitor PJ34 improved DNA binding of Sp1 and led to a dramatic increase in PARP-1 promoter function.

**Conclusion:**

Our results therefore recognized Sp1 as a target protein of PARP-1 activity, the addition of polymer of ADP-ribose to this transcription factor restricting its positive regulatory influence on gene transcription.

## Background

Poly(ADP-ribose) polymerase-1 (PARP-1) is a highly evolutionary preserved, multifunctional nuclear enzyme that catalyzes the addition of ADP-ribose (ADPr) units from nicotinamide adenine dinucleotide (NAD+) on a large variety of nuclear proteins and consequently impinges on many of the major nuclear functions (reviewed in [1]). Gene inactivation studies have revealed and/or confirmed up to nine biological functions for PARP-1 [2]. These include both DNA repair and maintenance of genomic integrity as well as regulation of telomerase activity [3,4] (also reviewed in [5]). PARP-1 also regulates the expression of various proteins at the transcriptional level (reviewed in [2]) and is also involved in DNA replication as well as cell differentiation [6-8]. In addition, polymer of ADP ribose (PAR) has been recently identified as an emergency source of energy used by the base-excision machinery to synthesize ATP [9,10]. PAR may also serve as a signal to induce cell death [11]. Finally, PARP-1 may contribute to the regulation of cytoskeletal organization and in the post-transcriptional modification of nuclear proteins like histones and transcription factors (reviewed in [2,12]).

PARP-1 plays vital functions in gene transcription as it can influence the state of chromatin remodeling through the catalytic addition of PAR to the core histones [13,14]. Besides, PARP-1, through its double Zn-finger DNA binding domain, can interact with target regulatory elements present in the promoter of many genes, including the MCAT 1 [15], Pax-6 [16], MHC II [17], and the CXCL1 [18] genes. By exploiting both chromatin cross-linking and immunoprecipitation assays, Soldatenkov and coworkers elegantly demonstrated that PARP-1 could also bind to secondary hairpin-like structures present in the 5'-flanking region of the human PARP-1 promoter [19]. Although no prototypical sequence that could be recognized as a target by the PARP-1 DBD has been identified yet, Pion and collaborators have however demonstrated the ability of the protein to bind to both 5'- and 3'-recessed ends on double-stranded DNA, as well as to palindromic-like structures often present in DNA [20]. Lastly, PARP-1 can also alter gene transcription through its ability to poly(ADP-ribosyl)ate several transcription factors, such as YY1 [21], NFKB [22], TFIIF [23], Oct-1 [24], B-MYB [25] and AP-2 [26], thereby preventing their binding with their specific promoter target sites [27].

The studies conducted over the last few decades have been mostly dedicated to the study of the many biological and cellular functions of PARP-1. Unfortunately, not so many studies have explored the molecular mechanisms that regulate the transcription of PARP-1 gene expression. Unlike its enzymatic activity, PARP-1 gene transcription is not activated by DNA strand breaks [28,29]. On the other hand, PARP-1 gene expression appears related to cell proliferation rather than DNA synthesis as PARP-1 mRNA has been shown to be more abundant in the G1 phase of the cell cycle [30-32]. To date, the PARP-1 promoter has been cloned from three different mammalian species: human [33], rat [34] and mouse [35]. All three mammalian basal promoters share structural similarities typical of housekeeping genes in that they lack a functional consensus TATA box, possess a high content of GC-residues, and bear a consensus initiator sequence (Inr) that overlaps the transcription initiation site. The human promoter has been shown to contain binding sites for the transcription factors Sp1, AP-2 [33], YY1 [21], and Ets [36], whereas the mouse promoter was recently shown to be down-regulated by a complex of adenovirus E1A protein and pRb [35]. Studies that we conducted on the rat PARP-1 (rPARP-1) proximal promoter have shown that its activity is primarily, but not entirely dependent on the recognition of five GC-rich binding sites (F1, F2, F3, F4 and US-1) by the positive transcription factors Sp1 and Sp3 [37,38]. Other transcription factors, such as those that belong to the Nuclear Factor I (NFI) family of transcription factors [39], were also found to bind to nearby target sites to alter the activity directed by the PARP-1 gene promoter [39-41]. Sp1 and Sp3, two members from a Zn-finger family of transcription factors that presently comprises nine proteins (Sp1 to Sp9) [42], often compete with each other for the recognition of their common GC-rich target sites [43,44]. However, Sp1 is a transcriptional activator, while Sp3 can, according to the context, function either as a repressor or an activator of gene transcription [43,45]. The NFI family is composed of four members encoded by distinct genes, NFI-A, -B, -C, and -X [46], producing distinct protein products, which can form homo- or heterodimers [47]. In addition, all four NFI mRNAs can be differentially spliced to yield a large number of NFI isoforms with subtle differences in their transactivation properties [48,49]. NFI has been reported to repress the activity directed by the rPARP-1 promoter [39,40]. However, as NFI was found to be transcriptionally inert by itself as it possesses no intrinsic activity in the regulation of the rPARP-1 promoter, its negative influence was suggested to result from the fact that it competes with Sp1 for the availability of a promoter composite element that bears overlapping target sites for both these transcription factors [40]. Consequently, by exploiting either the synergistic or antagonistic effects of the transcription factors they bind to respectively over-activate or down-regulate transcription, the combination of regulatory elements that constitute the PARP-1 promoter provides an efficient way of fine-tuning its expression in different cellular contexts.

As with many other nuclear proteins, both Sp1 and NFI can be subjected to post-translational modifications through mechanisms such as phosphorylation and/or glycosylation [50,51]. However, neither of these transcription factors, which are both required to ensure proper transcription of the PARP-1 gene, has been shown to be also subjected to poly(ADP-ribosyl)ation by PARP-1. In the present study, we examined both the expression and DNA binding properties of Sp1 and NFI in embryonic fibroblasts cultured from normal mice that express the intact, wild-type PARP-1 protein (PARP-1^+/+^), or from PARP-1 knockout mice (PARP-1^-/-^) that are devoided of any PARP-1 enzymatic activity [52]. We demonstrated that PARP-1 physically associates, through protein-protein interaction, with Sp1 but not Sp3 or NFI in immunoprecipitation assays. Sp1 was also found to be a target of PARP-1 as addition of PAR to this transcription factor could be demonstrated. Preventing poly(ADP-ribosyl)ation of Sp1 in the PARP-1 deficient knockout cells also resulted in a substantial increase in the activity directed by rat PARP-1 promoter, a clear indication that addition of PAR to Sp1 reduces its transactivation properties *in vivo*.

## Results

### Expression and DNA binding of Sp1, Sp3 and NFI in PARP-1^+/+ ^and PARP-1^-/- ^cells

**Figure 1:**
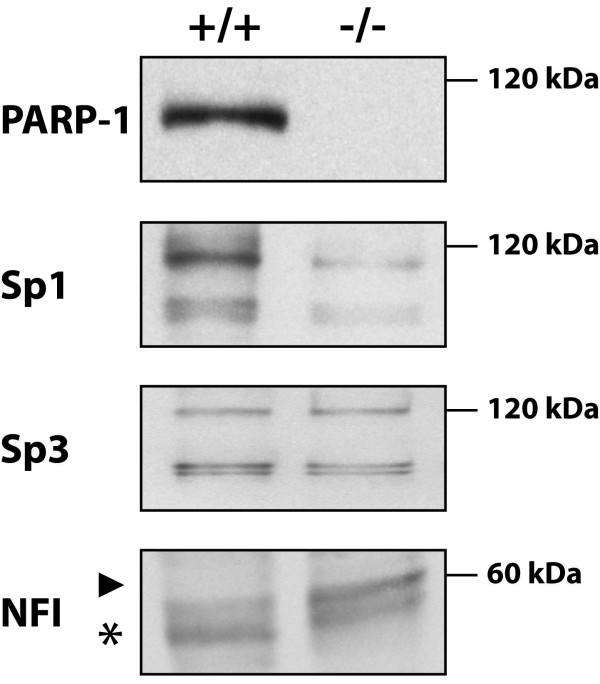
**Expression of PARP-1, Sp1, Sp3 and NFI in PARP-1^+/+ ^and PARP-1^-/- ^cells**. Crude nuclear extracts (10 μg) from both PARP-1^+/+ ^and PARP-1^-/- ^cells were examined in Western blot using antibodies directed against PARP-1, Sp1, Sp3 and NFI. The position of the 120 kDa and 60 kDa proteins used as molecular mass markers is indicated. The asterisk indicates the position of the typical NFI complex whereas the arrowhead designates NFI complexes with a reduced electrophoretic mobility that predominated in the extract from PARP-1^-/- ^cells. Data of one from three similar experiments are presented.

To investigate whether suppression of PARP-1 activity would result in alterations in the level of expression and/or the DNA binding activity of the transcription factors that are critical for the transcription of the PARP-1 gene, crude nuclear extracts were prepared from embryonic fibroblast cell lines derived from both normal (PARP-1^+/+^) and PARP-1 knockout mice (PARP-1^-/-^) [52]. These extracts were then used to monitor the expression and DNA binding activity of transcription factors (Sp1, Sp3 and NFI) that have been reported to play critical functions in PARP-1 gene transcription [37,39-41,53,54]. As expected, PARP-1^+/+ ^fibroblasts appropriately expressed the PARP-1 protein whereas no PARP-1 expression could be detected in the PARP-1^-/- ^cell line (Figure 1). Whereas no alteration was observed in the level of Sp3 expression between both types of cells, a dramatic reduction was however observed for Sp1 in PARP-1^-/- ^cells. NFI, which yields multiple bands in the EMSA due to the fact that different isoforms have been recognized for this transcription factor, primarily appeared as a fast migrating protein species when nuclear extracts from PARP-1^+/+ ^cells are used in Western blotting (* in Figure 1). However, the electrophoretic mobility of this NFI protein species was considerably reduced in PARP-1^-/- ^cells (arrowhead in Figure 1), suggesting that NFI is either subjected to post-translational modifications or to the differential expression of a specific isoform that is distinct from that seen in PARP-1^+/+ ^cells.

**Figure 2:**
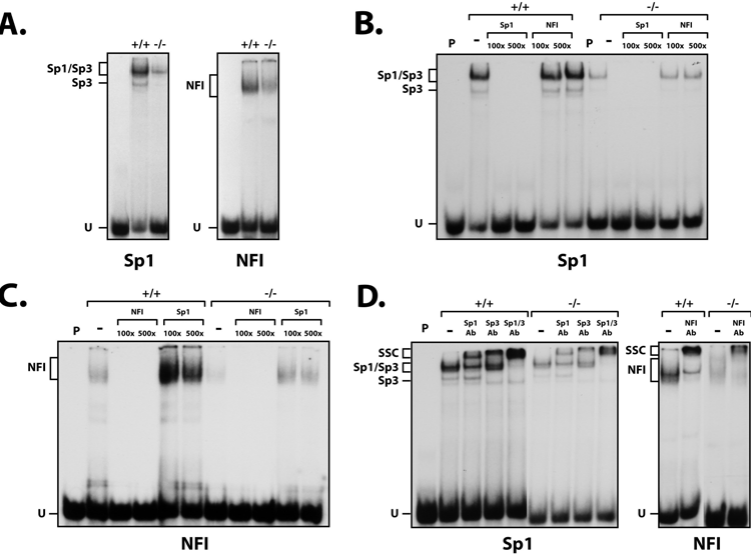
**DNA binding properties of Sp1/Sp3 and NFI in PARP-1^+/+ ^and PARP-1^-/- ^cells**. (**A**) EMSA analysis of Sp1/Sp3 and NFI. Crude nuclear proteins (5 μg) from both PARP-1^+/+ ^and PARP-1^-/- ^cells were incubated with a 5' end-labeled probe bearing the high affinity binding site for either Sp1 (left) or NFI (right). Formation of DNA/protein complexes was then monitored by EMSA on an 8% (Sp1) and 10% (NFI) native polyacrylamide gel and their position revealed through autoradiography. The position of both the Sp1/Sp3 and NFI DNA-protein complexes are shown, as well as that of the free probe (U). P: labeled probe alone. (**B**) Sp1 competition experiment in EMSA. The Sp1 labeled probe used in panel A was incubated with nuclear proteins (5 μg) from both PARP-1^+/+ ^and PARP-1^-/- ^cells in the presence of either no (-) or 100- and 500-fold molar excesses of unlabeled competitor oligonucleotides (either Sp1 or NFI). Formation of DNA/protein complexes was then monitored by EMSA on an 8% native gel. (**C**) NFI competition experiment in EMSA. Same as in panel B except that the NFI double-stranded oligonucleotide was 5'-end labeled and used as probe for the assay. (**D**) Supershift experiment in EMSA. Crude nuclear proteins from both PARP-1^+/+ ^and PARP-1^-/- ^cells were incubated with the either the Sp1 (5 μg proteins were used) or NFI (10 μg proteins were used) labeled probe in the presence of either no (-), or 2 μl of a polyclonal antibody directed against Sp1 (Sp1Ab) or Sp3 (Sp3Ab) and added either individually or in combination (Sp1+Sp3Ab) (left), or with a polyclonal antibody directed against NFI (right). Formation of both the Sp1/Sp3 and NFI complexes, as well as their corresponding supershifted complexes (SSC) is indicated. P: labeled probe alone; U: unbound fraction of the labeled probe.

In order to verify whether both the decrease in Sp1 expression and change in the mobility of the NFI protein also resulted in a corresponding alterations in the DNA binding of both transcription factors, EMSA experiments were conducted. Crude nuclear proteins from both the PARP-1^+/+ ^and PARP-1^-/- ^cells were therefore incubated with 5'-end labeled, double-stranded oligonucleotides bearing the high affinity-binding site for either Sp1 or NFI. DNA-protein complexes were resolved on native polyacrylamide gels and their position revealed by autoradiography. As shown on Figure 2A, both the NFI and Sp1 labeled probe yielded the appropriate DNA-protein complexes corresponding to the binding of both factors to their respective target sequence when incubated with the extract from PARP-1^+/+ ^cells. However, and consistent with the results of the Western blot analyses shown on Figure 1, formation of both complexes was considerably reduced when the Sp1 and NFI labeled probe were incubated with the extract from PARP-1^-/- ^cells. Formation of the Sp1 complex observed with the extracts from both the PARP-1^+/+ ^and PARP-1^-/- ^cells was found to be specific as it was entirely competed off by as little as a 100-fold molar excess of the unlabeled Sp1 oligomer, but not at all by the unrelated NFI site (Figure 2B). The reduced binding of Sp1 in PARP-1^-/- ^cells did not result from changes in the affinity of Sp1 toward its GC-rich target site but rather from a decrease in its expression at the protein level as a corresponding reduction was also observed for this transcription factor in Western blot analysis (Figure 1). Identical results were observed also for the NFI complex whose formation was competed by the NFI but not the Sp1 oligomers (Figure 2C).

The identity of the proteins that yielded the shifted complexes using the Sp1 labeled probe was further investigated by supershift analyses in EMSAs using antibodies directed against either Sp1 and Sp3, two closely related transcription factors from the same family [42]. As shown on Figure 2D, the Sp1 Ab strongly reduced the migration of the complex with the lowest electrophoretic mobility but had no influence on the weaker, faster migrating complex. On the other hand, the antibody against Sp3 only slightly decreased the electrophoretic mobility of the slow complex but entirely competed the fast migrating one. Incubation of both the Sp1 and Sp3 Abs together entirely supershifted both complexes, using the nuclear extracts from both the PARP-1^+/+ ^and PARP-1^-/- ^cell lines. Both antibodies yielded super-shifted complexes (SSC) corresponding to the signal yielded by the Ab-Sp1(Sp3)-DNA complex. These results indicate that the slow migrating complex is made up of both Sp1 and Sp3 bound to the labeled probe (primarily Sp1) whereas the faster migrating complex is entirely made up of Sp3. A similar experiment was also conducted on the DNA-protein complex yielded by the incubation of both the PARP-1^+/+ ^and PARP-1^-/- ^nuclear extracts with the NFI probe. As presented on Figure 2D, addition of the NFI Ab reduced the electrophoretic mobility of the NFI complex from both the PARP-1^+/+ ^and PARP-1^-/- ^protein extracts and yielded a new super-shifted complex resulting from the recognition of the NFI-DNA complex by the NFI Ab. We therefore conclude that expression and DNA binding of both Sp1 and NFI is severely reduced in the PARP-1 knockout (PARP-1^-/-^) cells.

**Figure 3:**
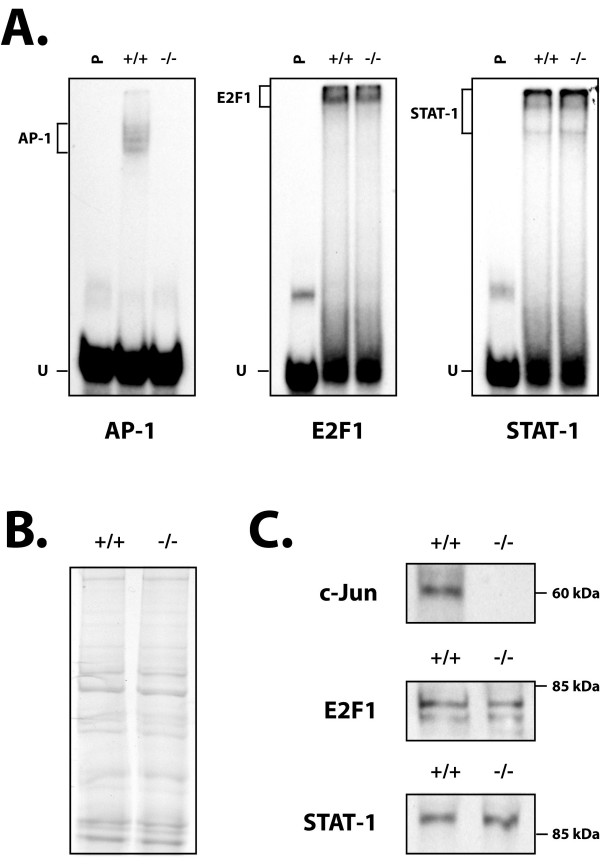
**DNA binding and expression of the transcription factors AP-1, E2F1 and STAT-1 in PARP-1^+/+ ^and PARP-1^-/- ^cells**. (**A**) EMSA analysis. Crude nuclear proteins (5 μg) from both PARP-1^+/+ ^and PARP-1^-/- ^cells were incubated with a 5' end-labeled probe bearing the high affinity binding site for AP-1, E2F1 and STAT-1. Formation of DNA/protein complexes was then monitored by EMSA on an 8% gel as detailed in Figure 2. The position of the AP-1, E2F1 and STAT-1 DNA-protein complexes is shown, as well as that of the free probe (U). P: labeled probe alone. (**B**) Coomassie blue staining of the protein samples used for conducting both the EMSA (panel A) and the Western blot experiment (panel C). One protein band present in both extract was randomly selected and its intensity determined by densitometric analysis in order to precisely calibrate the protein concentration used for the assays. (**C**) Nuclear extracts (10 μg) from both PARP-1^+/+ ^and PARP-1^-/- ^cells were examined in Western blot as in Figure 1 using antibodies directed against E2F1, STAT-1 and the AP-1 subunit c-jun. The position of the nearest molecular mass markers is indicated (60 kDa and 85 kDa).

To eliminate the possibility that a widespread suppression of all transcription factors might have accounted for the drop in both Sp1 and NFI expression in PARP-1^-/- ^knockout cells, we investigated the expression and DNA binding properties of transcription factors unrelated to the transcription of the PARP-1 gene. Double-stranded oligonucleotides bearing the target sites for the transcription factors AP-1, E2F1 and STAT-1 were therefore 5'-end labeled and used in EMSA in combination with nuclear extracts obtained from both PARP-1^+/+ ^and PARP-1^-/- ^cells. As shown on Figure 3, whereas binding of AP-1 to its prototypical target site was entirely abolished in PARP-1^-/- ^cells, that of both E2F1 and STAT-1 remained unaffected by the lack of PARP-1 expression in the knockout cells. Western blot analyses indicated that the lack of AP-1 binding resulted from the absence of c-jun in the PARP-1^-/- ^cells, whereas no change was observed in the expression of both the E2F1 and STAT-1 proteins. Therefore, the reduction in the expression and DNA binding of both Sp1 and NFI is closely linked to the lack of functional PARP-1 in the PARP-1^-/- ^knockout cells and is not merely the consequence of a general shutdown of gene expression that might have occurred in these cells.

### PARP-1 physically interacts with Sp1

**Figure 4:**
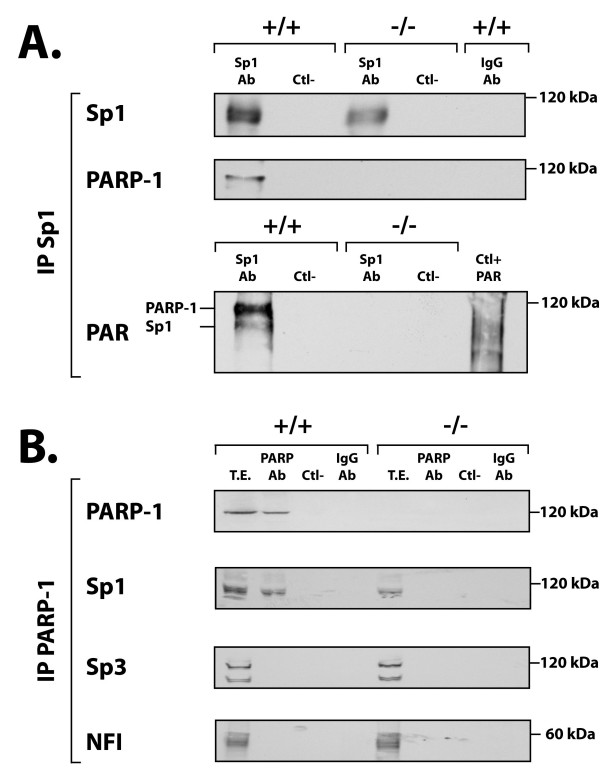
**Co-immunoprecipitation of Sp1 and PARP-1 in protein extracts from PARP-1^+/+ ^and PARP-1^-/- ^cells**. (**A**) Immunoprecipitation of the Sp1-protein complexes in PARP-1^+/+ ^and PARP-1^-/- ^nuclear extracts. Crude nuclear proteins (300 μg) from both PARP-1^+/+ ^and PARP-1^-/- ^cells were incubated with the Sp1 Ab (sc-59) and the Sp1-protein complexes recovered by the addition of protein-A-Sepharose. The resulting immunoprecipitated proteins were then SDS-gel fractionated before being membrane-transferred and Western blotted with antibodies against Sp1, PARP-1 (C-2-10) and PAR (LP-9610). Ctl-: protein A-Sepharose added to crude nuclear proteins in the absence of Sp1 Ab and used as a negative control. IgG-Ab: normal rabbit IgG incubated with nuclear proteins prior to addition of protein A-Sepharose as a negative control. (**B**) Immunoprecipitation of the PARP-1-protein complexes in PARP-1^+/+ ^and PARP-1^-/- ^nuclear extracts. Same as in panel A except that the immunoprecipitation was conducted using the PARP-1 F-123 Ab. The blotted, PARP-1-immunoprecipitated proteins were then analyzed with the PARP-1 (422), Sp1 (sc-59), Sp3 (sc-644), and PAR (LP-9610) antibodies. Negative controls (Ctl- and IgG-Ab) are as in panel A. TE: total cell extract that has not been immunoprecipitated with the PARP-1 Ab.

As PARP-1 has been shown to physically interact with many nuclear proteins of which some are transcription factors, we then wished to determine whether any of Sp1, Sp3 or NFI could represent a target for PARP-1 *in vitro*. Sp1 was therefore immunoprecipitated from nuclear extracts prepared from PARP+/+ and PARP-/- cells using the Sp1 Ab (sc-59) and the immunoprecipitated proteins analyzed on Western blot with Abs directed against either Sp1 or PARP-1 (C-2-10). As shown on Figure 4A, Sp1 was very efficiently immunoprecipitated with the Sp1 Ab as it could be detected in the extracts from both the PARP-1^+/+ ^and PARP-1^-/- ^cells. As expected, a much weaker Sp1 signal was obtained with the PARP-1^-/- ^nuclear extract. Western blotting the Sp1-immunoprecipitated proteins with the PARP-1 Ab revealed clearly the presence of PARP-1 in the extract from PARP-1^+/+ ^cells but not in that from PARP-1^-/- ^cells, suggesting that indeed, Sp1 and PARP-1 can physically interact with each other. As expected, no signal was observed when either protein A-Sepharose (Ctl-) or a rabbit IgG Ab were added as negative controls to the extract in the absence of Sp1 Ab. Blotting of the membrane with the LP-9610 Ab against PAR revealed the presence of two poly(ADP-ribosyl)lated proteins in the extract from PARP-1^+/+ ^but not PARP-1^-/- ^cells: a more intense band with an electrophoretic mobility identical to that corresponding to PARP-1, and a weaker, faster-migrating band with a mobility on gel identical to that expected for Sp1. Again, both negative controls (protein A-Sepharose (Ctl-) and rabbit IgG Ab) yielded no signal at a position similar to those seen with the Sp1 Ab. As a positive control, total proteins were prepared from *E. coli *cells transformed with a recombinant plasmid that encodes high levels of a fully functional, truncated PARP-1 [55], and used in Western blotting. As shown on Figure 4A, blotting of the bacterially produced recombinant PARP-1 protein with the PAR Ab LP-9610 revealed a smear that is typical of PAR-modified proteins [56].

To further validate the results from the Sp1 immunoprecipitation, the reverse experiment was conducted by first immunoprecipitating the PARP-1 associated proteins from total protein extracts prepared from PARP-1^+/+ ^and PARP-1^-/- ^cells using the F1–23 Ab against PARP-1. We selected this Ab primarily because it is much more efficient in immunoprecipitating PARP-1 than the C-2-10 Ab. The transcription factors Sp1, Sp3 and NFI could be detected easily by Western blot in the total extracts from both PARP-1^+/+ ^and PARP-1^-/- ^cells prior to immunoprecipitation of PARP-1 (T.E. in Figure 4B). Immunoprecipitation of PARP-1 with the F1–23 Ab and further Western blotting of the precipitated proteins revealed clearly that the F1–23 Ab was efficient in immunoprecipitating PARP-1 in the extract from PARP-1^+/+ ^but not from PARP-1^-/- ^cells. In addition, Sp1 could also be revealed in the immunoprecipitate using the sc-59 Ab. On the other hand, the Abs against Sp3 and NFI could not detect any of these transcription factors in the PARP-1 immunoprecipitate. We therefore conclude that Sp1 but not Sp3 nor NFI associate physically with PARP-1 *in vitro *through protein-protein interactions.

### PARP-1 enzymatic activity is not required for its interaction with Sp1

**Figure 5:**
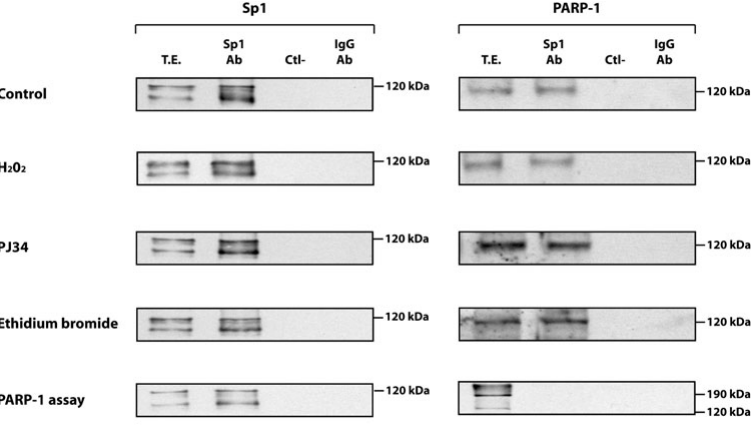
**Influence of PARP-1 activity on the co-immunoprecipitation of PARP-1 by Sp1**. Nuclear proteins (300 μg) from PARP-1^+/+ ^cells grown either alone (control) or in the presence of hydrogen peroxide (H_2_O_2_), PJ34 PARP-1 inhibitor, or ethidium bromide were incubated with the Sp1 Ab (sc-59) and the Sp1-protein complexes recovered by the addition of protein-A-Sepharose. The resulting immunoprecipitated proteins were then gel fractionated as in Figure 4 and Western blotted with antibodies against Sp1 or PARP-1 (C-2-10). TE: total cell extract that has not been immunoprecipitated with the Sp1 Ab. Ctl-: protein A-Sepharose added to crude nuclear proteins in the absence of Sp1 Ab and used as a negative control. IgG-Ab: normal rabbit IgG incubated with nuclear proteins prior to addition of protein A-Sepharose as a negative control.

We next examined whether the interaction between PARP-1 and Sp1 would be affected by the activation state of the PARP-1 enzyme in wild-type PARP-1^+/+ ^cells. Sp1 was therefore immunoprecipitated in nuclear extracts from PARP-1^+/+ ^cells that have been grown either alone (control), or in the presence of hydrogen peroxide (H_2_O_2_) to induce PARP-1 activity. Exposure of cells to H_2_O_2 _*in vivo *did not prevent nor improve the interaction between Sp1 and PARP-1 (Figure 5). Similarly, preventing PARP-1 activity by culturing PARP-1^+/+ ^cells in the presence of the PARP-1 inhibitor PJ34 did not alter the Sp1/PARP-1 interaction *in vivo*. The IP experiments shown above were all conducted on extracts obtained from PARP-1^+/+ ^cells in the absence of any added DNA. Although we could not detect any contaminating genomic DNA when increasing amounts (up to 10 μg) of the extract from the PARP-1^+/+ ^cells are ran on an agarose gel and then stained with ethidium bromide (data not presented), yet the possibility remained that undetectable traces of genomic DNA might have been present in our extracts and then contributed to the interaction between both Sp1 and PARP-1 by providing potential target sites to which Sp1 would first interact with. Therefore, in order to avoid any possible interference by any traces of contaminating genomic DNA, we repeated the IP experiment with the Sp1 antibody, but this time added ethidium bromide to the reaction mixture prior to the addition of the primary Abs in order to avoid any possible interaction of Sp1 with any putative target sites in DNA. As shown on Figure 5, the presence of ethidium bromide did not alter in any way the co-immunoprecipitation of PARP-1 along with Sp1 in PARP-1^+/+ ^cells. Most interestingly, over-activation of PARP-1 activity in *in vitro *PARP assays not only resulted in a massive addition of PAR to PARP-1, as revealed by the dramatic change in its electrophoretic mobility, but also entirely abolished interaction with Sp1 (Figure 5) therefore indicating that addition of PAR to PARP-1 beyond a certain level also interferes with its ability to bind Sp1.

### Sp1 is a target of PARP-1 enzymatic activity

**Figure 6:**
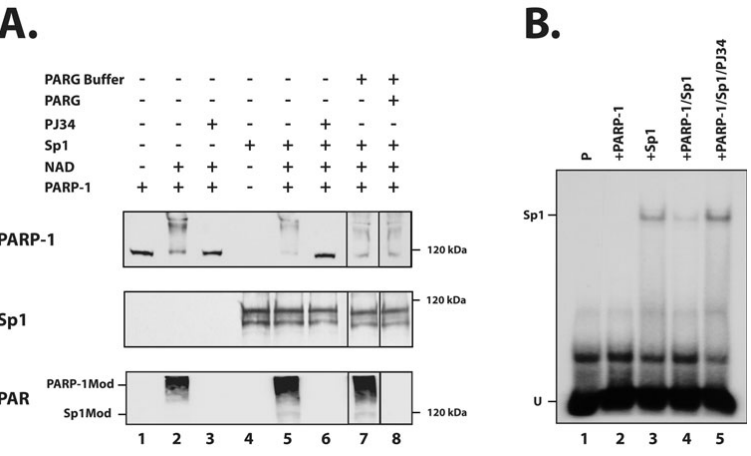
**PARP-1-dependent poly(ADP-ribosyl)ation of Sp1 *in vitro***. (**A**) Recombinant Sp1 protein was incubated in reaction buffer either alone (lane 4) or with purified bovine PARP-1 (1 unit) in the presence of 200 μM NAD+ (lane 5). The reaction mixture was subjected to Western blot analysis with the PARP-1 (C-2-10), Sp1 (sc-59) and PAR (LP-9610) antibodies. When indicated, the PARP inhibitor PJ34 was added to the reaction mixture with purified PARP-1 alone (lane 3) or in the presence of recombinant Sp1 (lane 6). When indicated, samples from the *in vitro *PARP assay were electrophoresed and electrotransfered onto nitrocellulose membranes. The PAR covalently linked onto the automodified PARP-1 and Sp1 proteins was then erased by incubation with PARG and the proteins analyzed by Western blotting with the same antibodies as detailed above (lane 8). Lane 1: PARP-1 alone; lane 2: PARP-1 incubated with NAD+; lane 3: same as in lane 2 plus PJ34; lane 7: same as in lane 5 but incubated in PARG buffer without addition of PARG-1. The position of modified PARP-1 (PARP-1Mod) and Sp1 (Sp1Mod) is indicated (left) along with the appropriate molecular mass marker (right). (**B**) Recombinant Sp1 was incubated in reaction buffer containing 200 μM NAD+ and nicked DNA either alone (+SP1; lane 3) or with purified bovine PARP-1 (1 unit) (+Sp1/PARP-1; lane 4). A sample (16 μl) from the reaction mixture was then incubated with the 5'-end labeled Sp1 oligonucleotide and formation of DNA-protein complexes monitored by EMSA as in Figure 2. As a control, the PARP-1 inhibitor PJ34 was added to the reaction mixture containing PARP-1/NAD^+^/Sp1 (+Sp1/PARP-1/PJ34; lane 5). Lane 1: labeled probe alone in reaction mix (P); Lane 2: labeled probe incubated in buffer D with PARP-1 but in the absence of NAD and Sp1 (+PARP-1). The position of both the Sp1 complex (Sp1) and the free probe (U) is indicated.

As the PAR Ab revealed the presence of a poly(ADP-ribosyl)ated protein in the Sp1 immunoprecipitate that has the same electrophoretic mobility as that of Sp1, we therefore used *in vitro *PARP assays to determine whether PARP-1 could add PAR to this transcription factor. Western blot analyses were conducted to monitor both the PARP-1 and Sp1 proteins, as well as their poly(ADP-ribosyl)ation by the addition of PAR. PARP-1 alone possesses no intrinsic activity in the absence of its substrate NAD^+ ^(Figure 6A, lane 1). However, the addition of 200 μM NAD^+ ^was sufficient to turn on PARP-1 activity, which then added PAR to itself (Figure 6A; PARP-1Mod: bottom panel) through its automodification domain. Automodification of PARP-1, which dramatically changed its electrophoretic mobility in SDS-PAGE (yielding a smear on the gel; Figure 6A, lane 2: top panel), was entirely prevented when the PARP-1 inhibitor PJ34 was added to the reaction mix (Figure 6A, lane 3). As shown on lane 4, recombinant Sp1 is initially free of PAR. However, upon incubation with both PARP-1 and NAD^+^, a faint band with a molecular mass corresponding to Sp1 (Sp1Mod) could be detected with the PAR Ab just beneath the signal (smear) corresponding to the automodified PARP-1 (Figure 6A, lane 5: bottom panel). Again, the addition of the PJ34 PARP-1 inhibitor entirely prevented the addition of PAR to both proteins (PARP-1 and Sp1; lane 6). In order to demonstrate the specific addition of PAR to both Sp1 and PARP-1, both recombinant proteins were incubated together along with NAD^+ ^as in lane 5. Protein samples were electrophoresed on SDS-PAGE and transferred on membranes as above. The PAR covalently linked onto the automodified PARP-1 and Sp1 proteins transferred on the membranes was then erased by incubation with PARG. As shown on Figure 6A (lane 8: bottom panel), no polymer could be detected by the PAR Ab on both the PARP-1 and Sp1 proteins upon exposure to PARG. The fact that the proteins were first fractionated on the SDS gel and membrane-transferred prior to the treatment with PARG explains the lack of any change in the electrophoretic mobility of PARP-1 on gel (lane 8, top panel), which again appears as a smear due to the initial addition of PAR.

**Figure 7:**
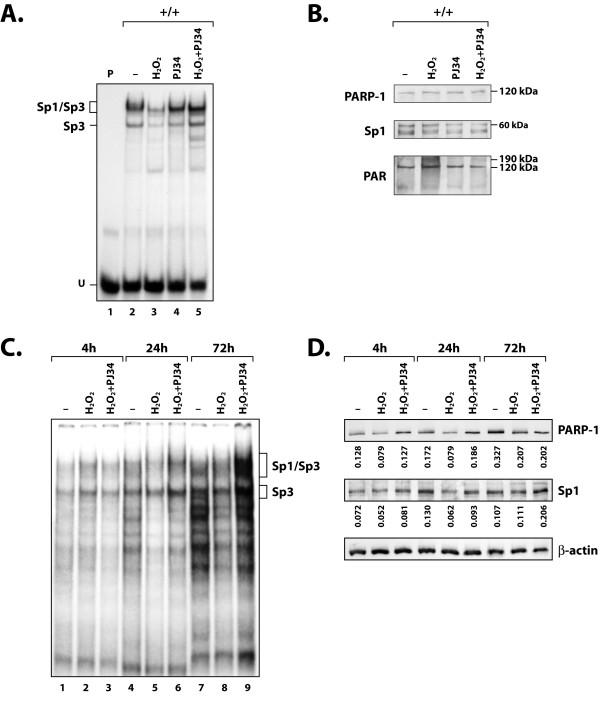
**In vivo influence of PARP-1 activity on the expression and DNA binding of Sp1**. (**A**) Nuclear proteins (5 μg) from PARP-1^+/+ ^cells grown alone (-; lane 2) or in the presence of H_2_O_2 _(lane 3) or PJ34 (lane 4), added either individually or in combination (PJ34+ H_2_O_2_; lane 5), were incubated with the Sp1 labeled probe and formation of DNA/protein complexes monitored by EMSA on a 8% native polyacrylamide gel as in Figure 2. The position of both the Sp1 and Sp3 DNA-protein complexes are shown, as well as that of the free probe (U). P: labeled probe alone (lane 1). (**B**) The extracts used in panel A were SDS-gel fractionated before being membrane-transferred and Western blotted with antibodies against Sp1 (sc-59), PARP (C-2-10) and PAR (10-H). The position of the appropriate molecular mass markers (60-, 120-, and 190 kDa) is indicated. (**C**) Nuclear proteins (5 μg) from primary cultures of HSKs grown for various periods of time (4-, 24- and 72 h) either alone (-; lanes 1, 4 and 7), or in the presence of H_2_O_2 _(lanes 2, 5 and 8) or both H_2_O_2 _and PJ34 (PJ34+ H_2_O_2_; lanes 3, 6 and 9), were incubated with the Sp1 labeled probe and formation of DNA/protein complexes monitored by EMSA on a 8% native polyacrylamide gel as in panel A. (**D**) The extracts used in panel C were analyzed by Western blotting with antibodies against Sp1 (sc-59), PARP (C-2-10) and β-actin (CLT9001). Densitometric analyses of the band intensities was determined for both the Sp1 and PARP-1 proteins and normalized to that measured for β-actin. Values are shown below each corresponding track.

We then monitored whether addition of PAR to Sp1 *in vitro *would alter its DNA binding properties in EMSA. As shown on Figure 6B (lane 3), the recombinant Sp1 protein yielded the typical Sp1 DNA-protein complex upon addition of the Sp1 labeled probe when incubated with the reaction mix containing both NAD^+ ^and nicked DNA in the absence of PARP-1. However, addition of PARP-1 to the reaction mix severely reduced binding of Sp1 to its corresponding labeled probe (by approximately 71%, as revealed by densitometric analysis of the labeled DNA-protein complex)(Figure 6B, lane 4). The further addition of the inhibitor PJ34 prevented the addition of PAR by PARP-1 and entirely restored the binding properties of Sp1 (Figure 6B, lane 5). We next tested whether the addition of PAR to endogenous Sp1 would also reduce its DNA binding properties *in vivo *in crude nuclear extracts prepared from PARP-1^+/+ ^cells grown in the presence of H_2_O_2 _to activate PARP-1 in these cells. A dramatic reduction in the binding of Sp1 to its DNA target probe was observed in EMSA when PARP-1 activity was increased in H_2_O_2_-treated cells (Figure 7A, compare lane 3 with lane 2). This H_2_O_2_-dependent reduction in Sp1 binding was entirely abolished when H_2_O_2_-treated cells were also added the PARP-1 inhibitor PJ34 (PJ34+ H_2_O_2_; Figure 7, lane 5). Western blot analyses provided evidence that neither of these treatments (addition of PJ34 or H_2_O_2_) had any influence on the PARP-1 or Sp1 protein level (Figure 7B), a further indication that the reduced binding of Sp1 in H_2_O_2_-treated cells can be accounted for by post-translational addition of PAR to Sp1 by PARP-1. Monitoring the addition of PAR to the PARP-1 protein with the 10-H antibody against the polymer confirmed that exposing PARP-1^+/+ ^cells to the PARP-1 inducer H_2_O_2 _indeed resulted in the automodification of endogenous PARP-1, that again appears as a smear in Western blotting (PAR; Figure 7B).

There are many potential hypotheses that may explain why expression of both endogenous Sp1 and PARP-1 does not change upon incubation with PJ34. However, and as the cells treated with PJ34 were harvested only 18 hours later, we initially thought they might not have had sufficient time to alter their pattern of protein expression. We therefore conducted a new set of experiments but selected primary cultures of human skin keratinocytes (HSKs) as a cellular model for PARP-1 expression rather than PARP-1^+/+ ^cells. HSKs were first exposed to H2O2 in order to trigger activation of endogenous PARP-1 in these cells, and then harvested at various periods of times (4-, 24- and 72 hours) for further analysis of Sp1 DNA binding and both Sp1 and PARP-1 expression at the protein level. As an additional control, cells triggered with H2O2 were also added PJ34 at each selected time-point prior to harvesting. Neither H2O2 nor H2O2+PJ34 had any significant influence on the DNA binding properties of Sp1 at 4 h of treatment (Figure 7C; compare lane 1 with lane 2), which is also supported by the weak influence of these reagents on both Sp1 and PARP-1 protein expression in Western blot (Figure 7D). However, challenging PARP-1 activity through exposure of HSKs to H2O2 not only reduced the binding capacity of Sp1 (and to some extend, Sp3 as well) in these cells at 24 and 72 h (Figure 7C) but also translated into a reduction in the expression of both PARP-1 and Sp1 proteins (Figure 7D). Most interestingly, the H2O2-dependent reduction in the DNA binding of both Sp1 and Sp3 was not solely reverted by the further addition of the PARP-1 inhibitor PJ34 but was even considerably improved at 72 h (Figure 7C; compare lane 9 with lane 8). This dramatic increase in Sp1 DNA binding also translated in an increased expression of Sp1 at the protein level, which nearly doubled upon addition of the PJ34 inhibitor (ratio of the Sp1/β-actin signal of 0.111 with H2O2 and 0.206 with H2O2+PJ34, as revealed by densitometric analysis of each protein band). We therefore conclude that PARP-1-dependent addition of PAR to Sp1 reduces its DNA binding properties and thereby alter the efficiency with which it can transcribe its target genes, which comprise the PARP-1 gene besides from the Sp1 gene itself.

### Influence of poly(ADP-ribosyl)ation on the activity directed by the rPARP-1 promoter

**Figure 8:**
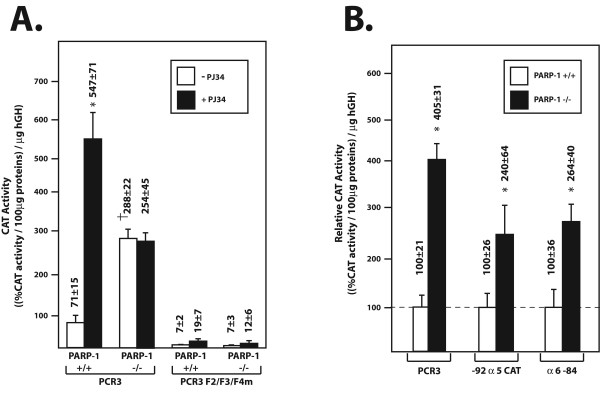
**rPARP-1 promoter activity in PARP-1^+/+ ^and PARP-1^-/- ^cells**. (**A**) The recombinant plasmids PCR3 and PCR3F2/F3/F4m were transfected into both PARP-1^+/+ ^and PARP-1^-/- ^cells grown with or without the PARP-1 inhibitor PJ34. CAT activities were measured and normalized to the amount of hGH secreted into the culture medium. Values are expressed as ((%CAT activity/100 μg proteins)/ng hGH). Asterisks (*) indicate CAT activities from cells exposed to PJ34 that are statistically different from those measured when cells are transfected with pCR3 in the absence of inhibitor whereas † corresponds to CAT activities in PARP-1^-/- ^cells that are statistically different from those measured in PARP-1^+/+ ^cells (*P *< 0.005; paired samples, *t*-test). S.D. is also provided. (**B**) The plasmids PCR3, -92α5CAT and α6–84 were transfected into both PARP-1^+/+ ^and PARP-1^-/- ^cells and CAT activity (expressed as the ratio of CAT activity from PARP-1^-/- ^cells over that measured in PARP-1^+/+ ^cells (considered as 100%)) measured and normalized as detailed above. Asterisks (*) correspond to CAT activities in PARP-1^-/- ^cells that are statistically different from those measured in PARP-1^+/+ ^cells (*P *< 0.005; paired samples, *t*-test).

Most of the activity directed by the PARP-1 gene promoter was shown to rely essentially on the recognition of multiple PARP-1 promoter target sites by members of the Sp1 family [37,38,41]. Of them, Sp1 accounted for most of the positive influence exerted on this promoter [54]. We demonstrated above that both expression and DNA binding of Sp1 is considerably decreased in PARP-1^-/- ^cells. We therefore examined whether this decrease would also translate into a reduced PARP-1 promoter activity upon transfection of PARP-1^-/- ^cells with a recombinant construct bearing the CAT reporter gene fused to the basal promoter from the rat PARP-1 gene that has its three Sp1 target sites (F2, F3 and F4) either kept intact (in pCR3) or mutated (in pCR3F2/F3/F4m) [37]. Unexpectedly, transfection of the pCR3 construct into PARP-1^-/- ^cells yielded CAT activities approximately 4-fold higher than in PARP-1^+/+ ^cells (Figure 8A). Consistent with these results, exposing pCR3-transfected PARP-1^+/+^cells that express the wild type PARP-1 protein to the PARP-1 inhibitor PJ34 resulted in a near 8-fold increase in basal PARP promoter activity while it had no influence in PARP-1^-/- ^cells. Mutating all three Sp1 sites from the PARP-1 promoter (in pCR3F2/F3/F4m) dramatically reduced basal promoter activity and entirely suppressed induction of PARP-1 promoter function in PARP-1^-/- ^cells. Suppression of PARP-1 activity with PJ34 in PARP-1^+/+ ^cells had no influence on the activity directed by pCR3F2/F3/F4m suggesting that the regulatory influences of PARP-1 on its own promoter are mediated through alteration of Sp1.

As the trans-activating properties of the PAR-free Sp1 is increased in PARP-1^-/- ^cells, we then wished to determined whether the transcriptional activity directed by other gene's promoter reported to be under the regulatory influence of this transcription factor would also be similarly affected by the lack of PARP-1 activity. We recently reported that transcription of the human α5 integrin subunit gene was under the positive regulatory influence of both a proximal and a distal Sp1 site in primary cultures of rabbit corneal epithelial cells [57,58]. We therefore used a recombinant construct that bear the CAT reporter gene fused to a truncated version of the α5 promoter that contain only the proximal Sp1 site (-92α5CAT) for conducting this experiment. In addition, and as the GC-rich promoter from the human α6 integrin subunit gene was also reported to be positively regulated by Sp1 [59,60], a recombinant construct that has the CAT gene fused to the basal promoter from the α6 gene (α6–84) was also used in these assays. The -92α5CAT and α6–84 constructs were therefore transfected into both the PARP-1^-/- ^and PARP-1^+/+ ^cell lines and CAT activity determined and normalized. As shown on Figure 8B, transfection of the PARP-1 promoter-bearing construct pCR3 yielded a CAT activity that was more than 4-times more elevated in PARP-1^-/- ^than in PARP-1^+/+ ^cells (which is expressed as the ratio of the activity measured in PARP-1^-/- ^over that from PARP-1^+/+ ^cells). Interestingly, transfection of both the α5 and α6 promoter constructs also yielded increased CAT activities in PARP-1^-/- ^cells (2.4- and 2.6-fold increases, respectively), indicating that the altered regulatory influence resulting from the lack of PAR addition to Sp1 in PARP-1^-/- ^cells is not merely restricted to the PARP-1 promoter but also affect transcription of other Sp1-responsive genes as well.

## Discussion

The ability of the PARP-1 protein to interact with a variety of nuclear-located transcription factors and thereby alter their regulatory function toward the target genes that they regulate is certainly among the most important of the many functions PARP-1 may play as it will ultimately alter the pattern of genes expressed by any given cell. Although an increasing number of such transcription factors are being reported every year, yet only a few of them have been shown to be post-translationally modified by PARP-1. Here we show that PARP-1 physically associates with the positive transcription factor Sp1 and reduces its trans-activating properties by the catalytic addition of PAR to this protein. We demonstrate that such a post-translational alteration of Sp1 not only reduces the transcription of the PARP-1 gene itself (as the lack of PARP-1 activity in PARP-1^-/- ^cells also translates into a more than 4-fold increase in PARP-1 promoter function, a result consistent with those reported by Soldatenkov et al. [19]), but also that of other Sp1-responsive genes, for instance those encoding the human integrin subunits α5 and α6. Based on the fact that the average branching frequency of the PAR is approximately one branch per linear section of 20–50 units of ADPr [61-63], and that ADPr units have a molecular mass of 577 Daltons, we can assume that PAR branching will translate in an increase of at least 11.5 kDa in the molecular mass of the acceptor protein. The unchanged apparent electrophoretic mobility of Sp1 in Western blot suggests that branching of ADPr units is however unlikely to occur. This is consistent with poly(ADP-ribosyl)ation of other transcription factors, namely RAP30/RAP74 subunits of TFIIF [23]. An analysis of the 24 potential poly(ADP-ribosyl)ation sites (glutamic acids residues)in Sp1 (Accession no NP_612482) reveals that 8 of them are localized within the first 140 amino acids (N-terminal) whereas 12 are localized at the C-terminal end of the protein. Most of the sites are in hydrophilic portions of the protein (20 out of 24) and therefore available to accept PAR. The two last sites at position E752 and E757 are in an hydrophobic portion and might not be readily modified by PAR. Modification by PAR anywhere on the protein may induce its desorption from DNA. However, two potential sites (E672 and E690) are localized within the second and third Zn finger of Sp1, respectively. The specific targeting of these sites may be critical in negatively regulating the function of Sp1 on specific DNA sequence.

Sp1 modification by PAR alters the expression of the PARP-1 gene because of the presence of at least five distinct target sites for this transcription factor in the PARP-1 gene promoter [37]. Besides its action on Sp1, PARP-1 has also been reported to regulate the transcription of its own gene through other regulatory mechanisms that are most likely related to the presence of secondary structures in DNA. Indeed, it has been shown that the DBD of PARP-1 can repress PARP-1 gene transcription by interacting directly with hairpin-like structures present in the promoter of the human PARP-1 gene [19]. Although these results may appear contradictory at first sight to those we report in this study, yet the possibility remains that the PARP-1 truncated version they used might have caused repression of the PARP-1 promoter through the recruitment of other transcription factors rather than through a direct action of PARP-1 on Sp1. For instance, PARP-1 has been reported to physically interact with the transcription factors TEF-1, B-MYB and AP-2 [15,16,25,26,64]. Interestingly, all three transcription factors have been shown to function not only as activators but also as repressors of gene transcription [65-69]. Besides, the two most likely hairpin-like structures in the human PARP-1 promoter have been shown to be present at quite a distance from the Sp1 target sites-bearing basal promoter (from nt -325 to -290 and -418 to -403). It is also worth mentioning that mutation of all three Sp1 sites contained in the rPARP-1 basal promoter entirely suppressed the induction of PARP-1 promoter activity in PARP-1^-/- ^cells, a clear evidence that the PARP-1 negative influence on its own promoter-driven transcription is entirely dependent on altering Sp1 binding at the rPARP-1 promoter.

Addition of PAR to transcription factors can either promote or reduce their affinity toward their corresponding sites in DNA. It has been shown that the DNA binding properties of a few transcription factors, notably YY-1, NFkB, CREB, the TFIID subunit TBP, and presumably Sp1, was severely reduced in the presence of NAD+ [27]. The pattern of migration that they obtained for Sp1 on the EMSA was, however, not typical of that normally observed for this transcription factor and might have resulted either from protein degradation of the crude nuclear extracts used by these authors or from the fact that a transcription factor other than Sp1 has bound to the GC-rich labeled probe they used for conducting their EMSA. Sp1 belongs to a large family of transcription factors, the Sp/KLF family, which comprises 25 proteins that all possess the ability to bind GC- or GT-rich target sites in DNA as all of them share three similar Zn-fingers as their DNA binding domain [70]. As no supershift experiments using an anti-Sp1 antibody were conducted in the study by Oei and coworkers, care must be taken as to whether Sp1 indeed was the transcription factor from the HeLa extract that actually bound their labeled probe in EMSA as the shifted band might have resulted from the binding of another member from the Sp/KLF family to the GC-rich labeled probe they used.

The experiments that we conducted either *in vitro*, using purified Sp1, or *in vivo*, by inducing the PARP-1 activity with the oxidative reagent H_2_O_2_, provided evidence that addition of PAR to Sp1 not only lowered its positive regulatory influence on gene transcription but also its capacity to physically interact with its high affinity target sites in DNA. This exposition of cultured cells (for instance HSKs) to H_2_O_2 _also translated in a reduced expression of both PARP-1 and Sp1 (as Sp1 was reported to regulate the transcription of its corresponding gene through the presence of multiple, functional GC-rich target sites [71,72]) at the protein level, a process that could be reverted in a time-dependent manner by the PARP inhibitor PJ34 (Fig. 7). It is important to note that PJ34 has a broad range, and therefore inhibits the activity of not only PARP-1 but other PARPs as well. This would include PARP-2, a known DNA-dependent PARP involved in DNA damage response and repair [73,74]. However, because PARP-1 accounts for more than 90% of cellular poly(ADP-ribosyl)ation [75], it makes the contribution of PARP-2, or other PARPs, marginal on modification of Sp1. Moreover, the use of PARP-1-/- cells strongly support the *in vivo *role of PARP-1 in the modulation of Sp1 activity. That no reduction, but rather an increase in the expression of Sp1 is seen in PARP-1^+/+ ^cells (Figs. 1 and 2) is rather paradoxical with the results from the transfection experiments. Yet, the lack of any PARP-1 influence when all three Sp1 sites from the PARP-1 promoter-bearing construct PCR3 are mutated is a clear evidence that the primary action of PARP-1 in wild-type PARP-1^+/+ ^cells is directed toward this transcription factor. As an interesting hypothesis, we suggest that in PARP-1 expressing cells, most, if not all Sp1 proteins has been added PAR to a certain level, which would represent a remarkable mean for the cell to restrict the otherwise very powerful trans-activating properties of Sp1 on gene transcription. Although the basal level of Sp1 expression is decreased in PARP-1^-/- ^knockout cells whereas that of other transcription factors, such as Sp3, E2F1 and STAT-1, remains unaffected, it would however be free of added PAR and would thus become a much more potent activator of gene expression. Besides reducing its trans-activating properties, addition of PAR to Sp1 may also serve other cellular functions. Sp1 has been recently reported as particularly sensitive to protein degradation in quiescent, post-confluent, but not in actively growing primary cultures of rabbit corneal epithelial cells (RCECs) [57]. Monitoring the steady state level of Sp1 in cells grown in the presence of PARP-1 inhibitors such as 3-AB or PJ34 in cells grown at a high cell density might prove particularly informative considering the recent demonstration that PARP-1 contributes to the activity of the proteasome in drug-induced, oxydatively damaged nuclear proteins [76-78]. Abnormally elevated expression and DNA binding of Sp1, as for many of the Sp/KLF family members to which belong Sp1 [79], has been recognized as a valuable prognostic marker in both gastric [80-82] and colorectal cancers [83]. PARP-1 mediated addition of PAR to Sp1 can now be viewed as one out of a few other post-translational modifications, such as glycosylation and phosphorylation, that the cell may use to modulate the positive regulatory influence of Sp1 and thereby contribute to prevent any given cell to progress toward anchorage-independent, unregulated cell proliferation, the hallmark of all cancer cells.

**Figure 9:**
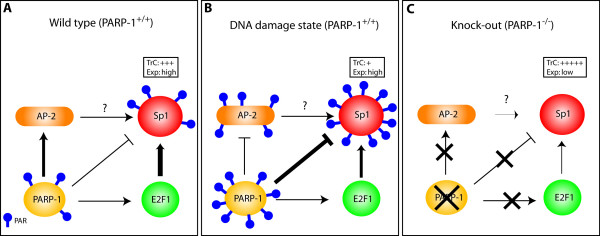
**Model of interplay between PARP-1, Sp1 and other transcription factors**. (**A**) PARP-1 plays a suppressive function (indicated by 'T' bars) on the DNA binding properties of Sp1, and indirectly, on its expression as well, by the enzymatic addition of poly(ADP-ribose) units (PAR) to Sp1. PARP-1 may exert its effect by stimulating the transcriptional properties (indicated by arrows) of both AP-2 and E2F-1 by physically interacting with these transcription factors (and therefore, independently of addition of PAR), of which the latter was recognized as a component required to ensure proper transcription of the human Sp1 gene. (**B**) Once PARP-1 is stimulated by DNA damages, post-translational modification of both Sp1 and AP-2 is increased to the point that their DNA binding properties and thereby, their transcriptional capacity, is considerably decreased without significantly altering their level of expression. (**C**) However, in the absence of PARP-1, addition of PAR is abrogated and the transcriptional capacity of Sp1 becomes dramatically increased despite that its overall expression is considerably reduced primarily as a consequence of: i) a reduction in both the expression [112] and the positive transcriptional influence of E2F1 [85], a property that requires a physical interaction with PARP-1, and ii) a reduced transcriptional activity of AP-2, which also requires a physical association of this transcription factor with the middle region of PARP-1 [84]. TrC: transcriptional capacity of Sp1; Exp: level of Sp1 expression.

Although the reduced binding of Sp1 in wild-type PARP-1^+/+ ^cells grown in the presence of the PARP activator H_2_O_2 _(Fig. 7A) is easily explained by the capacity of PARP-1 to add PAR to this transcription factor, the similar reduction observed in Sp1 expression (Fig. 1) and DNA binding (Fig. 2A) between both wild-type PARP-1^+/+ ^and PARP-1^-/- ^knockout cells remains speculative. However, the recent cloning of the human Sp1 gene promoter raises interesting possibilities to highlight the influence the lack of PARP-1 protein might have on the reduced expression of Sp1 (refer to Fig. 9 for additional details). Indeed, the basal promoter of the human Sp1 gene was found to bear target sites for multiple transcription factors, including NF-Y, AP-2, E2F, C/EBP as well as Sp1 itself [71]. Interestingly, the transcriptional activity directed by both AP-2 and E2F1 was found to be regulated by PARP-1 activity in cultured cells. PARP-1 apparently exerts a dual regulatory influence on AP-2α transcription with opposing effects: the middle region of the PARP-1 protein interacts physically with AP-2α and enhance its transcription, a situation postulated to occur under normal circumstances, whereas the catalytic domain strongly poly(ADP-ribosyl)ates AP-2α and thereby reduces binding to its DNA target sites, a temporary shut-off mechanism that might be used during unfavourable conditions [84]. On the other hand, E2F-1 was not found to be added PAR by PARP-1 but was found to physically interact with it, which results in an improved binding of E2F-1 to its DNA target sites and further enhances its trans-activating properties, and indicating that PARP-1 acts as a positive co-factor of E2F-1-mediated transcription [85]. Although binding of AP-2 to the Sp1 gene promoter could not be demonstrated, E2F was found to bind and positively influence transcription of that gene [71]. Therefore, the reduced expression of Sp1 that we observed in PARP-1^-/- ^knockout cells may simply result from the reduced transcriptional activity of E2F-1 (and maybe also of AP-2) as the Sp1 gene has been reported to be positively regulated in a dose-dependent manner by this transcription factor [71] (Fig. 9).

Besides Sp1, our results also demonstrated a reduced expression of the transcription factors AP-1 and NFI in PARP-1^-/- ^knockout cells relative to wild type in PARP-1^+/+ ^cells, whereas that of others, such as Sp3, E2F1 and STAT-1 remained unaffected by the lack of PARP-1 activity. That AP-1 expression and DNA binding is strongly abrogated in PARP-1 deficient cells is a well documented fact [86-90]. Indeed, the pharmacological inhibition of PARP-1 activity in 3,4-dihydro-5-[4-(1-piperinidinyl)butoxyl]-1(2H)-isoquinoline (DPQ)-treated mice entirely abolished the TPA-dependent activation and DNA binding properties of AP-1 [86]. Inhibition of PARP-1 activity in rats treated with nicotinamide was found to prevent N-methyl-N-nitrosourea-induced photoreceptor apoptosis through a reduction in the expression of the AP-1 subunit c-jun, a process that was postulated to depend on the inhibition of the JNK/AP-1 signalization pathway [87]. PARP-1 activity was recently shown to be required for c-jun phosphorylation, a process that modulate the stability of AP-1 and determines its DNA binding efficiency [90]. DNA binding of NFI was found to be strongly abrogated in the PARP-1^-/- ^knockout cells. These alterations in the DNA binding properties of NFI also correlated with important changes in the pattern of NFI protein electrophoretic mobility on SDS gels that might result either from the expression of distinct NFI isoforms, or from post-translational modifications of NFI that may differ between the wild-type PARP-1^+/+ ^and PARP-1^-/- ^knockout cells. To our knowledge, no study has ever reported such a suppression of the NFI DNA binding properties in PARP-1^-/- ^knockout cells. We previously demonstrated that NFI has a negative influence on the activity directed by the rPARP-1 promoter but not because it possesses any intrinsic repressor function but rather because it competes with Sp1 for the availability of a promoter composite element that bears overlapping target sites for both these transcription factors [39,40]. Interestingly, members from the NFI family have been reported to be as efficient as repressors [40,91,92] than activators [93,94] of gene transcription. Besides, NFI sites have often been shown to be located close to, or overlapping with nearby Sp1 sites. Indeed, NFI was reported to interact with and antagonize Sp1, which result in the down-regulation of the platelet-derived growth factor (PDGF)-A gene expression [95]. We recently reported that a similar interference of Sp1 action by NFI might also account for the transcriptional repression of the p21 genes [96]. The physiological significance of NFI suppression in the PARP-1^-/- ^knockout cells has yet to be precisely determined, although one would predict that its suppression would tend to favor the positive influence of the potentiated action of Sp1 that results from its lack of PAR addition.

## Conclusion

PARP-1 clearly contributes in a positive fashion to initiation of gene transcription by inducing local relaxation of the otherwise condensed chromatin by the attachment of PAR to the histones H1, H2A and H2B (reviewed in [97]). The recent demonstration that PARP-1 is closely associated to the enzyme topoisomerase IIβ (TopoIIβ [98]) further stressed the contribution of PARP-1 in the initiation of gene transcription as TopoIIβ, through its ability to create double-strand breaks in DNA, also provides a mean to activate PARP-1 enzymatic activity as well. Our results demonstrate that besides its positive action on gene transcription, PARP-1 may as well contribute to suppression of gene expression by its ability to add PAR to the strong transcriptional activator Sp1, thereby establishing a dual regulatory function (activation and repression) for this protein in gene expression.

## Methods

### Plasmids and oligonucleotides

The plasmid pCR3 as well as the pCR3 derivative plasmid that bear mutations in the three most proximal Sp1 binding sites (F2, F3 and F4; PCR3/F2F3F4m), have both been previously described [37]. The recombinant plasmids α6–84 which bear the chloramphenicol acetyltransferase (CAT) reporter gene fused to a DNA fragment covering the human α6 gene promoter sequence from positions -84 to +76 relative to the α6 mRNA start site was constructed as follow: The pGEM-3Z f(+) AvaI/α6 plasmid that bears the human α6 gene promoter from position -887 to +76 relative to the α6 mRNA start site (and generously provided by Schei Kitazawa, Kobe University School of Medicine, Kusunoki-Cho, Chuo-Ku, Kobe) was linearized at its unique KpnI site (5'-end) and treated with the nuclease Bal31 before being blunted. The 5'-digested α6 promoter fragments generated were then digested with HindIII (3'-end at α6 position +76) before being ligated into the unique SmaI/HindIII sites of the plasmid pGL3 to derive the pGL3/α6–590 vector. The pGL3/α6–590 construct was then digested with NheI and blunt-ended with Klenow (which then preserved the α6 -590 5'-end), before being second-digested with XbaI (which preserved the α6 +76 3'-end). The resulting α6 promoter-bearing DNA fragment was then ligated upstream the CAT reporter gene from the pCATBasic vector (Promega) that has been first digested with Pst I (5'-end), blunt-ended with Klenow and second digested with XbaI (3'-end) prior to its dephosphorylation with alkalin phosphatase (to yield pCATBasic/α6–590). Removal of the α6 promoter fragment from the common SphI site (located upstream from α6 promoter position -590 in the multiple cloning site of the parental plasmid pCATBasic) to the internal restriction site SacII (at α6 position -84) followed by blunt-ending with Klenow and ligation with T4 ligase, yielded the plasmid α6–84. The PXGH5 plasmid is a kind gift of Dr. David D. Moore (Department of Molecular and Cell Biology, Baylor College of medicine, Houston, TX, USA). The double-stranded oligonucleotides bearing the high affinity binding site for the transcription factors Sp1 (5'-GATCATATCTGCGGGGCGGGGCAGACACAG-3') [50], Stat-1 (5'-AAGGCGGAGGTTTCCGGGAAAGCAGCACC-3') [99], AP-1 (5'-GATCCCCGCGTTGAGTCATTCGCCTC-3') [100], E2F1 (5'-CAGAGCCGGCGGGAAAGGTGCGGGCGGTGC-3') [101] and NFI (5'-GATCTTATTTTGGATTGAAGCCAATATGAG-3') [102] were chemically synthesized using a Biosearch 8700 apparatus (Millipore, Bedford, MA, USA).

### Cell culture and media

The embryonic fibroblast cell lines derived from both normal (PARP-1^+/+^) and PARP-1 knockout mice (PARP-1^-/-^) [52] were grown in Dulbecco's modified Eagle's medium (DMEM) supplemented with 10% fetal bovine serum (FBS), and gentamycin (20 μg/mL). Human skin keratinocytes (HSKs) were isolated from a normal adult skin specimen (26 year-old donor) removed during reductive breast surgery and grown on a feeder layer of irradiated mouse Swiss 3T3 fibroblasts in complete keratinocyte medium as described [103]. Cells were maintained at 37°C, under a 5% CO_2 _controlled atmosphere in a humidified incubator. When indicated, both PARP-1^+/+ ^cells and HSKs were grown in the presence of either 300 μM hydrogen peroxide (H_2_O_2_) for 10 min, and/or 1 μM PARP-1 inhibitor PJ34 for either 24 h (PARP-1^+/+ ^cells) or for 4-, 24- and 72 h (HSKs); cells were also re-challenged (1 h prior to harvesting for PARP-1^+/+^cells, or every 24 h for HSKs) before they were harvested for the preparation of the nuclear extracts.

### SDS-PAGE and Western Blot

The protein concentration from the nuclear extracts prepared from PARP-1^+/+ ^and PARP-1^-/- ^cells was determined by the Bradford procedure and precisely validated through Coomassie blue staining on 10% SDS-polyacrylamide gels (a typical example can be seen on Figure 3B). Either 20 μg nuclear proteins was added to 1 volume of sample buffer (6 M urea, 63 mM Tris (pH6.8), 10% (v/v) glycerol, 1% SDS, 0,00125% (w/v) bromophenol blue, 300 mM β-mercaptoethanol) and then size-fractionated on a 10% SDS-polyacrylamide minigel before being transferred onto a nitrocellulose filter. The blot was then washed in TS (150 mM NaCl, 10 mM Tris-HCl pH7.4) and TSM buffers (TS buffer with 5%milk and 0,1% Tween 20) as described previously [58]. The membrane was further incubated for 1 h at 22°C with a 1:5000 dilution (except for the PARP-1 Ab (C-2-10) that has been used at a 1/10 000 dilution) of the following primary antibodies: rabbit polyclonal antibodies directed against the transcription factors Sp1 (sc-59; Santa Cruz Biotechnology, Inc. Santa Cruz, CA), Sp3 (sc-644; Santa Cruz Biotechnology), NFI (Santa Cruz Biotechnology, Inc.), c-jun (sc-44x; Santa Cruz Biotechnology), E2F1 (sc-193; Santa Cruz Biotechnology), and STAT-1 (G16920; Transduction Laboratories, BD Biosciences, Mississauga, CA), polyclonal Abs directed against a peptide from the C-terminal part of the PARP-1 automodification domain (422), or against PAR (LP-9610 or 10-H antibodies), or a mouse monoclonal antibody (C-2-10) raised against bovine PARP (both the 96-10 and C-2-10 Abs were bought from Dr. Guy Poirier, Unit of Health and Environment, CHUL Research Center, Québec, Canada). Membranes were also blotted with a mouse monoclonal antibody directed against β-actin (CLT9001, Jackson Immuno Research laboratories inc.; 1:35000 dillution), which has been used for normalization purposes. The blot was then washed and incubated with a 1:1000 dilution of a peroxidase-conjugated goat anti-rabbit (for the Sp1, Sp3, NFI, PARP-1 (422), p(ADP)r (LP-9610)) or anti-mouse (for the PARP-1 C-2-10 Ab) immunoglobulin G (Jackson Immunoresearch Lab.) and immunoreactive complexes revealed using a Western blot Detection Kit (Amersham, Baie d'Urfé, Canada). Each Western blot result shown in this study corresponds to one out of at least three representative experiments.

### Nuclear extracts and electrophoretic mobility shift assay (EMSA)

Crude nuclear extracts were prepared as described [104] from both the PARP-1^+/+ ^and PARP-1^-/-^cell lines and dialyzed against DNaseI buffer (50 mM KCl, 4 mM MgCl_2_, 20 mM K_3_PO_4 _(pH 7.4), 1 mM β-mercaptoethanol, 20% glycerol). Extracts were kept frozen in small aliquots at -80°C until use. EMSAs were carried out by incubating 3 × 10^4^cpm of 5' end-labeled (^32^P-γATP) double-stranded oligonucleotides bearing the high affinity binding sites for the transcription factors Sp1, NFI, AP-1, E2F1, and STAT-1 with either 5- (for the Sp1 probe) or 10 μg (for the NFI, AP-1, E2F1 and STAT-1 probes) nuclear proteins in the presence of 25 ng poly(dI-dC). poly(dI-dC) (Pharmacia-LKD) in buffer D (5 mM HEPES; 10% glycerol; 0,05 mM EDTA; 0,125 mM PMSF). When indicated, unlabeled double-stranded oligonucleotides bearing various DNA target sequences for known transcription factors (Sp1, NFI) were added as unlabeled competitors (100- and 500-fold molar excesses) during the assay. The EMSA experiment with the Sp1 from the *in vitro *PARP assay (16 μl from the reaction mix was used for each lane) was conducted as above except that poly(dI-dC) was omitted and that DNA-protein complexes were separated on a 6% native polyacrylamide gel. Supershift experiments in EMSA were conducted by adding antibodies (400 ng) directed against Sp1, Sp3 and NFI to the above reaction mixtures. Incubation proceeded at room temperature for 5 min upon which time DNA-protein complexes were separated by gel electrophoresis through a 8% (for Sp1, AP-1, E2F1 and STAT-1) or 10% (for NFI) native polyacrylamide gel run against Tris-glycine buffer as described [105]. Gels were dried and autoradiographed at -80°C to reveal the position of the shifted DNA-protein complexes.

### Immunoprecipitation assay

The immunoprecipitation of the Sp1 complexes was performed as follow: approximately 300 μg nuclear extracts from the PARP-1^+/+ ^and PARP-1^-/- ^cell lines was mixed with either the anti-Sp1 antibody (2 μg; Santa Cruz) or normal rabbit IgG (2 μg; Santa Cruz) overnight at 4°C, either alone, or in the presence of 100 μg/ml ethidium bromide [22,106], in binding buffer (10 mM Tris-HCl, 20 mM MgCl_2_, 150 mM NaCl, 1 mM dithiothreitol, 10% (v/v) glycerol, 1 mM PMSF). Protein-A-Sepharose (Sigma-Aldrich) was then added to the mixtures containing the cell extracts and incubated further for 5 h at 4°C. Samples were centrifuged and the protein A-Sepharose pellet washed four times in binding buffer before being resuspended in SDS sample buffer (6 M urea, 63 mM Tris (pH 6,8), 10% (vol/vol) glycerol, 1% SDS, 0,00125% (wt/vol) bromophenol blue, 300 mM β-mercaptoethanol). The immunoprecipitated samples were then used for Western blot analyses using the Sp1 (sc-59), PARP-1 (C-2-10) and PAR (LP-9610) antibodies. To immunoprecipitate the PARP-1 complexes, PARP-1^+/+ ^and PARP-1^-/- ^cells were cultured as described above and then harvested in lysis buffer (1% (v/v) NP-40, 175 mM KPO_4_, 150 mM NaCl, 1 mM dithiothreitol, 0,5 mM PMSF). The cell lysates were incubated for 1 h at 4°C and then cleared by centrifugation at 13,000 rpm for 10 min at 4°C. The anti-PARP-1 polyclonal antibody F-123 (100 μl) attached to protein A-Sepharose was then added to the cell lysates and the reaction mix incubated for 2 h at 4°C. The beads were pelleted by centrifugation, washed four times in lysis buffer and finally resuspended in SDS sample buffer. The immunoprecipitated samples were then used for Western blot analyses using the Sp1 (sc-59), Sp3 (sc-644), PARP-1 (422) and PAR (LP-9610) antibodies.

### In vitro Poly(ADP-ribosyl)ation assay

Recombinant Sp1 protein (250 ng GST-Sp1-8xHis protein; kindly provided by Dr. Claude Labrie, Oncology and Molecular Endocrinology Research Center, CHUL) was incubated for 5 min at 30°C with 1 U of purified bovine PARP-1 in a standard assay mixture (152 μl) containing 100 mM Tris-HCl, pH 8.0, 10 mM MgCl_2_, 10% (v/v) glycerol, 1.5 mM DTT, 10 μg/ml activated DNA (DNaseI treated) and 200 μM NAD^+ ^[107]. The reaction was terminated by the addition of SDS sample buffer (6 M urea, 63 mM Tris (pH 6,8), 10% (vol/vol) glycerol, 1% SDS, 0,00125% (wt/vol) bromophenol blue, 300 mM β-mercaptoethanol), and the resulting mixture subjected to Western blot analysis with the PARP-1 (C-2-10), Sp1 (sc-59) and PAR (LP-9610) antibodies. When indicated, the PARP inhibitor PJ34(Alexis Biochemicals, San Diego CA) was added to the reaction mixture at a final concentration of 100 mM along with purified PARP-1 15 min prior to the addition of the recombinant Sp1 protein.

### Erasable blot

Samples from the *in vitro *PARP assay were electrophoresed on 10% SDS-PAGE and electrotransfered onto nitrocellulose membranes at 50 V. The PAR covalently linked onto the automodified PARP-1 and Sp1 proteins transferred on the membranes was erased by incubation with poly(ADP-ribose) glycohydrolase (PARG) according to a previously described procedure [108]. The blots were subsequently soaked in TS (150 mM NaCl, 10 mM Tris-HCl pH7.4) containing 0,1% (v/v) Tween-20 and 5% non-fat milk (TSM) for 1 hour and then soaked in a solution of 50 mM KPO_4_, pH 7,4, 50 mM KCl, 5 mM β-mercaptoethanol, 10% (v/v) glycerol, 0,1% (v/v) Triton X-100 containing 2,5 U/mL of purified calf thymus PARG [109] for 2 hours at room temperature. Membranes were washed four times with 10 mL of renaturation buffer (150 mM NaCl, 10 mM Tris-HCl pH7.4) at 15 min intervals. The membranes were finally blotted with antibodies directed against PARP-1 (C-2-10), Sp1 (sc-59) and PAR (LP-9610).

### Transient transfection and CAT assay

PARP-1^+/+ ^and PARP-1^-/- ^cells were grown on tissue culture plates and transiently transfected 24 h later using the polycationic detergent Lipofectamine (Invitrogen-Gibco, On, Canada) following the procedure recommended by the manufacturer. When indicated, the PARP inhibitor PJ-34 (1 μM; Alexis Biochemicals) was added 3 h post-transfection. Each Lipofectamine-transfected plate received 1 μg of the PCR-CAT test plasmid and 1 μg pXGH5, which bears a secreted version of the human growth hormone gene upstream the mMT-I promoter [110]. Levels of CAT activity for all transfected cells were determined as described [111] and normalized to the amount of hGH secreted into the culture media and assayed using a kit for quantitative measurement of hGH (Immunocorp, Montréal, Québec). The value presented for each individual test plasmid transfected corresponds to the mean of at least three separate transfections done in triplicate. Student's t-test was performed for comparison of the groups. To be considered significant, each individual value needed to be at least three times over the background level caused by the reaction buffer used (usually corresponding to 0.15% chloramphenicol conversion). Each single value was expressed as 100 × (% CAT in 4 h)/100 μg protein/ng hGH. Differences were considered to be statistically significant at P < 0.05. All data are expressed as mean ± SD.

## Authors' contributions

KZ conducted all the experiments presented in this manuscript. She also contributed to the writing of the text.

SL conducted the experiments that yielded the results appearing on Figure 7C and 7D.

SD contributed intellectually to some aspects of the manuscript (especially those that describe the results contained in figures 4, 5 and 6) and participated to its writing.

SLG intellectually contributed to this study by designing the project. He also wrote most of the manuscript and revised its final version prior to submission.

All the authors read and approved the final manuscript.
